# Carbon coated Fe_0.65_Ni_0.30_Mn_0.05_ magnetically separable adsorbent for phenanthrene removal

**DOI:** 10.1038/s41598-025-13895-3

**Published:** 2025-08-29

**Authors:** Fagr A. Shehata, Mahmoud S. Abdel-Wahed, Mohamed Obaida, Amer S. El-Kalliny, Tarek A. Gad-Allah

**Affiliations:** 1 Water Pollution Research Department, National Research Centre, 33 El Buhouth St., Dokki, Giza, 12622 Egypt; 2 Physics Division, Solid State Physics Department, National Research Centre, 33 El Buhouth St., Dokki, Giza, 12622 Egypt

**Keywords:** Magnetic materials, Invar alloy, Water decontamination, Phenanthrene removal, Adsorption, Environmental sciences, Chemistry, Materials science, Nanoscience and technology

## Abstract

Fe_0.65_Ni_0.30_Mn_0.05_ invar alloy was produced via the polyol process, employing a polyvinyl alcohol (PVA) solution in varying weight ratios (50% and 66% PVA), and carbonized at different temperatures in argon (Ar) atmosphere to create a magnetic core-shell structure of Fe_0.65_Ni_0.30_Mn_0.05_@C. The structural, morphological, and magnetic characteristics of the prepared material were analyzed using X-ray diffraction (XRD), high-resolution transmission electron microscopy (HRTEM), Brunauer-Emmett-Teller (BET) method, Fourier-transform infrared spectroscopy (FTIR), and vibrating sample magnetometry (VSM). The carbonization process applied to these samples enhances their suitability for the adsorption of weakly polar or nonpolar organic molecules. The performance analysis of the synthesized magnetic adsorbent indicates that the sample prepared using 66% PVA, annealed at 550 °C , achieved the highest removal efficiency of 99.5% for phenanthrene as a model compound for polyaromatic hydrocarbons (PAHs) in aqueous media. The adsorption process described by the Temkin model is adept at exploring the relationship between indirect interactions among adsorbates and the adsorbent. This includes the initial interaction of adsorbate molecules with the most energetic sites, followed by subsequent interactions influenced by the presence of already adsorbed molecules. This is also confirmed by the compliance with the second-order kinetics. The optimum sample exhibits a superparamagnetic property with high saturation magnetism (22 emu/g), facilitating its recovery from treated water effluent via magnetic separation. Conclusively, the synthesized magnetic adsorbent materials show great potential as effective solutions for the remediation of water contaminated with PAHs.

**Supplementary Information:**

The online version contains supplementary material available at 10.1038/s41598-025-13895-3.

## Introduction

Polynuclear aromatic hydrocarbons (PAHs) are hydrophobic organic compounds composed of two or more fused aromatic rings. These compounds can be found in the environment either naturally (e.g., from forest fires and volcanic eruptions) or due to human activities (e.g., oil spills, industrial wastes, and effluents from various industries, including oil drilling, refining, coal tar processing, chemical manufacturing, and wood preservation)^1^. They were recognized as environmental pollutants in the late 1970s and early 1980s^2^. Most PAHs are known to have significant acute toxicity, carcinogenicity, teratogenicity, mutagenicity, and genotoxicity to various microorganisms, animals, and plants^3,4^. The concentrations of PAHs in wastewater effluents from municipal and industrial sources exhibit considerable variability based on their origin. For instance, urban stormwater runoff has been documented to contain total PAH levels of approximately 24 µg/L^5^, whereas industrial discharges, particularly from petrochemical and coking facilities, can present PAH concentrations ranging from 107 to 1707 ng/g-dw^5,6^. In municipal wastewater treatment facilities, influent PAH concentrations generally fall between 0.03 and 8,310,000 ng/L, although certain studies have indicated elevated levels, particularly in regions with substantial industrial activity^7^. To address the potential hazards linked to PAH pollution, regulatory bodies have implemented maximum contamination levels and environmental quality standards. The U.S. Environmental Protection Agency (EPA) has identified 16 PAHs as priority pollutants, establishing maximum contaminant goals (MCLGs) for specific compounds, such as benzo[a]pyrene, which is set at 0.2 µg/L in drinking water^8,9^. According to the EU Water Framework Directive (2008/105/EC, as amended), the annual average environmental quality standard (EQS) for benzo[a]pyrene is established at 0.002 µg/L, while the cumulative concentration of four priority PAHs (benzo[a]pyrene, benzo[b]fluoranthene, benzo[k]fluoranthene, and indeno[1,2,3-cd]pyrene) must not exceed 0.12 µg/L in surface waters^10^. Likewise, the World Health Organization (WHO) advises a guideline value of 0.01 µg/L for benzo[a]pyrene in drinking water^11^. Therefore, it is important to control PAH levels to minimize their negative impacts. The removal of PAHs from water has been the subject of extensive research using physical-chemical methods such as membrane filtration, coagulation, advanced oxidation processes, and adsorption^12–15^. Among these methods, adsorption stands out as a highly effective, economically viable, low energy demand, and environmentally friendly approach^16^particularly when using various adsorbent media like activated carbon, biochar, and modified clay minerals, which have demonstrated the ability to achieve up to 100% efficiency in removing PAHs from aqueous solutions^17^. Activated carbon, known for its cost-effectiveness, is a preferred choice for adsorption techniques^18^. Fine carbon powder, highly carbonaceous material, with a high porosity and sorption ability^19^, is an effective water treatment agent. However, recovering this type of carbon when used as a slurry in practical applications might be challenging. Although packing fine carbon powder in columns is an option, it requires pressurized influent, increasing the total cost. Therefore, we propose a magnetically separable carbon in core@shell form. The magnetic component, based on Invar alloy (Fe_0.65_Ni_0.35_), offers good corrosion resistance, toughness, ductility, and superparamagnetic character^20,21^. Adding 5% Mn to the common Fe_0.65_Ni_0.35_ invar alloy improves the magnetism^22^, and thermal stability^23^. Therefore, this alloy can be used as a magnetic core for adsorptive carbon (i.e., Fe_0.65_Ni_0.30_Mn_0.05_@C core@shell), which has not yet been developed or used so far as we know, for the removal of phenanthrene (PHE), one of the simplest PAHs containing three fused aromatic rings. Through the use of a magnetic component, the adsorbent can be separated, recovered, and reused from treated effluents using an externally applied magnetic field. This makes Fe_0.65_Ni_0.3_Mn_0.05_@C a versatile option, offering a high active surface area when used in slurry form.

One of the key aspects of this core-shell design is the thickness of the carbon layer, which plays a crucial role in influencing the magnetism of the core as well as the effective surface area. Careful control of the carbon layer thickness would result in the creation of more adsorptive centers, leading to enhanced adsorbent performance without compromising the magnetic affinity. This research presents the initial preparation of a magnetically modified Invar core Fe_0.65_Ni_0.30_Mn_0_._05_ utilizing the polyol method, which is recognized as an environmentally sustainable approach due to the employment of non-toxic ethylene glycol as both solvent and reducing agent. Polyvinyl alcohol (PVA) was selected as the carbon source for the creation of a hydrophobic adsorbent, owing to its inert, adhesive, cost-effective, non-toxic, and water-soluble characteristics^24^. The comprehensive characterization and performance evaluation focused on the removal of PHE, a representative compound for PAHs pollutants. Furthermore, the adsorption studies encompassed optimization, adsorption isotherm, kinetics, and the reuse of the prepared adsorbent, thus providing valuable insights into its efficacy.

## Experimental

### Materials

All materials and reagents were used as received. To prepare Fe_0.65_Ni_0.30_Mn_0.05_@C, the following chemicals were used: iron(II) chloride tetrahydrate (FeCl_2_·4H_2_O, Alpha Chemika), nickel(II) chloride hexahydrate (NiCl_2_·6H_2_O, Rasayan Laboratories), manganese(II) chloride tetrahydrate (MnCl_2_·4H_2_O, Prolabo), ethylene glycol (PioChem), sodium hydroxide (NaOH of high purity > 98%, Loba Chemie), and poly(vinyl alcohol) (Mw 9,000–10,000, 80% hydrolyzed (Sigma-Aldrich).

### Methodology

The Fe_0.65_Ni_0.30_Mn_0.05_ alloy was prepared using the polyol method. First, iron, nickel, and manganese salts were dissolved in 132 mL of ethylene glycol. Then, 7 g of NaOH was added, and the mixture was refluxed at 190 °C for 1 h. The resulting precipitate was recovered through centrifugation (MEGAFUGE 16, Thermo Fisher Scientific (10 min at 10000 rpm) and magnetic separation (Neodymium magnet (TM-30 × 50-N magnet, magnets4you Co., Germany). It was washed with double-distilled water, then ethanol, and finally dried under vacuum at 60 °C.

**Fig. 1:**
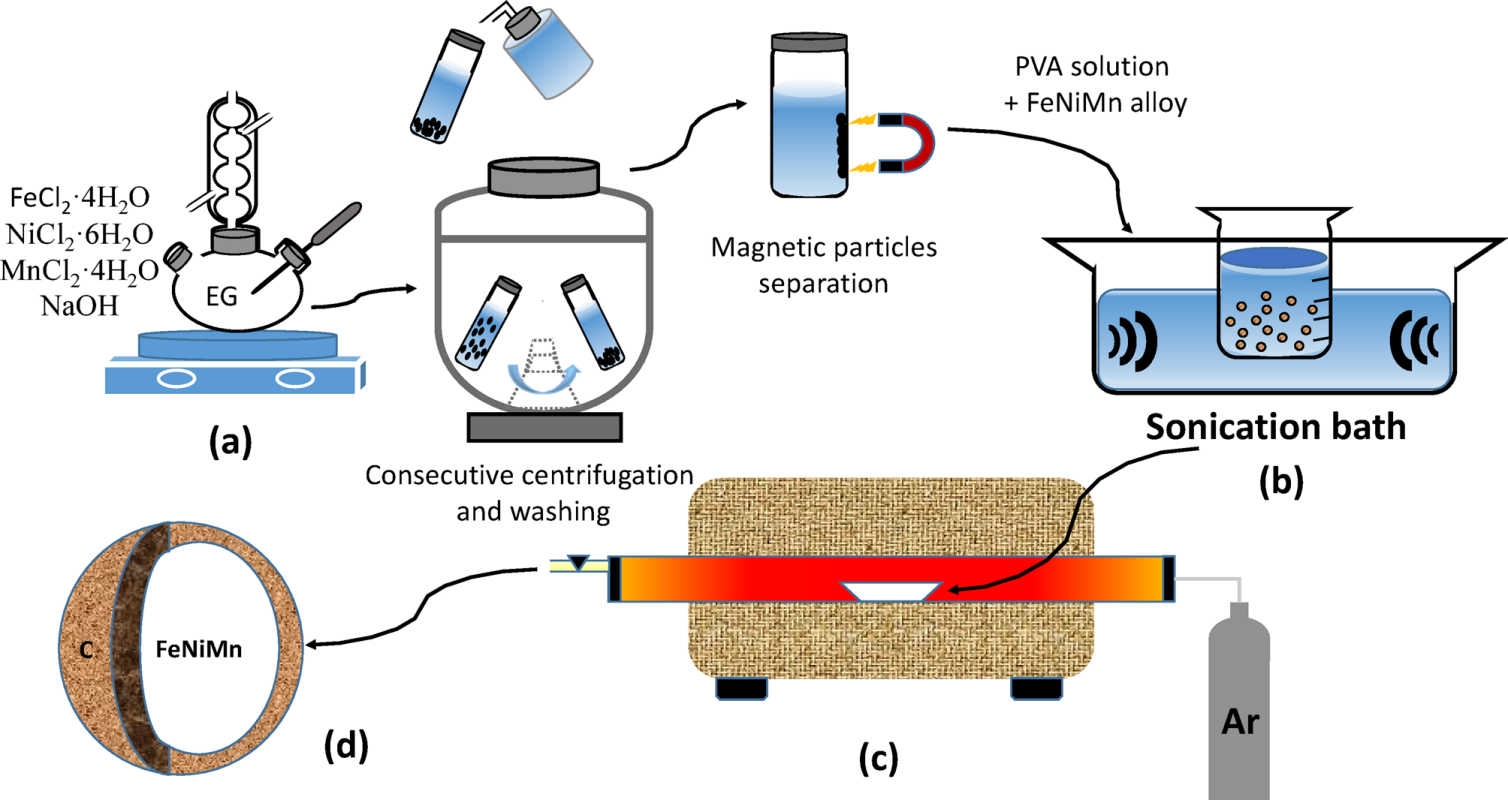
Schematic diagram representing the preparation steps of Fe_0.65_Ni_0.30_Mn_0.05_@C: (**a**) refluxing of the salts in EG, (**b**) Mixing of magnetic particles with PVA, (**c**) carbonization and annealing in a tube furnace, (d) production of the magnetic alloy@C.

The process involved carbon coating of the magnetic powder by mixing it with PVA solution using an ultrasonic cleaner (DAIHAN Scientific) for 10 min at different weight percentages of 50% and 66% (i.e., Fe_0.65_Ni_0.30_Mn_0.05_:PVA in 1:1 and 1:2 ratios, respectively). The alloy was carbonized and annealed simultaneously by heating the PVA/alloy mixture at various temperatures in a homemade tube furnace at different annealing temperatures of 350 °C, 450 °C, and 550 °C for 2 h under a continuous argon (Ar) flow to prevent oxidation. The furnace consists of a quartz tube (inner diameter 50 mm) and a PID-controlled heating system, allowing precise temperature control (± 1 °C). Samples were heated at a ramp rate of 5 °C/min up to 550 °C and held at the target temperature for 2 h before naturally cooling to room temperature under an argon atmosphere. The magnetic powder was then separated using a neodymium magnet to selectively recover the Fe_0.65_Ni_0.30_Mn_0.05_@C composite. The recovered powder was thoroughly washed with distilled water in an ultrasonic cleaner for 5 min to remove any loosely attached carbon particles. An illustrative preparation scheme is provided in Fig. 1.

The crystallinity of the prepared materials was analyzed using the X-ray diffraction (XRD) technique. A PANalytical X’Pert Pro diffractometer from the Netherlands, equipped with a CuKα source (λ = 1.5406 Å), was utilized for this purpose. The magnetic properties of the materials were determined from the hysteresis loops obtained using Riken Denshi BH-55 vibrating sample magnetometer (VSM). Functional groups were identified from the Fourier transform infrared (FT-IR) traces recorded using a Varian 3100 FT-IR Excalibur Series. The morphologies of the materials were examined using high-resolution transmission electron microscopy (HR-TEM) (JEOL TEM-2100, Japan). N_2_ adsorption-desorption isotherms were collected using BELsorp max (BEL Japan Inc.) surface analyzer. The specific surface areas and other texture characteristics were calculated based on the measured isotherms.

The PHE adsorption experiments using the prepared magnetic adsorbents were conducted in a batch system at a constant temperature of 27 ± 1 °C. In these experiments, the magnetic adsorbents were mixed with 100 mL of PHE solution at various concentrations. Prior to transferring the mixture to a shaking water bath (Wisebath, Wis Laboratory Instruments CO.), the suspensions underwent 20 s of sonication to ensure homogenization. The conical flasks were then agitated at 115 rpm. At specific time intervals, 5 mL of the suspension was withdrawn. Using a neodymium magnet, the adsorbent was separated before determining the PHE concentration in the supernatant using a UV-Visible spectrophotometer (JASCO V630, Japan). The PHE concentration was quantified by its absorbance at 250 nm with a six-point linear calibration curve of R^2^ = 0.9984 within the range of 0.05 mg/L–1.5 mg/L (Fig. S1). The removal efficiency (%) and the adsorbed amount of PHE ($\:{q}_{t}$, mg/g) were determined using the following formula:

1 $$\:Removal\:\left(\%\right)=\frac{\left({C}_{o}-{C}_{t}\right)\:}{{C}_{o}}\times\:100$$

2 $$\:{q}_{t}=\left({C}_{o}-{C}_{t}\right)\times\:\frac{\:V}{m}$$

where, $\:{C}_{o}$ (mg/L) and $\:{C}_{t}$ (mg/L) are the concentrations of PHE at the initial time and at certain times, respectively; $\:V$ (L) is the volume of the PHE solution, and $\:m$ (g) is the weight of the magnetic adsorbent.

The optimization of the adsorption process entailed a comprehensive exploration of the influences of contact time, initial pH, magnetic adsorbent dose, and initial PHE concentration. This involved the application of nonlinear curve fittings to ascertain the behavior of the adsorption isothermal models and adsorption kinetics, thereby precluding the pitfalls inherent in linear fitting^25–27^. The experimental conditions are provided within the figure captions.

## Results and discussion

### Characterization of the prepared materials

**Fig. 2:**
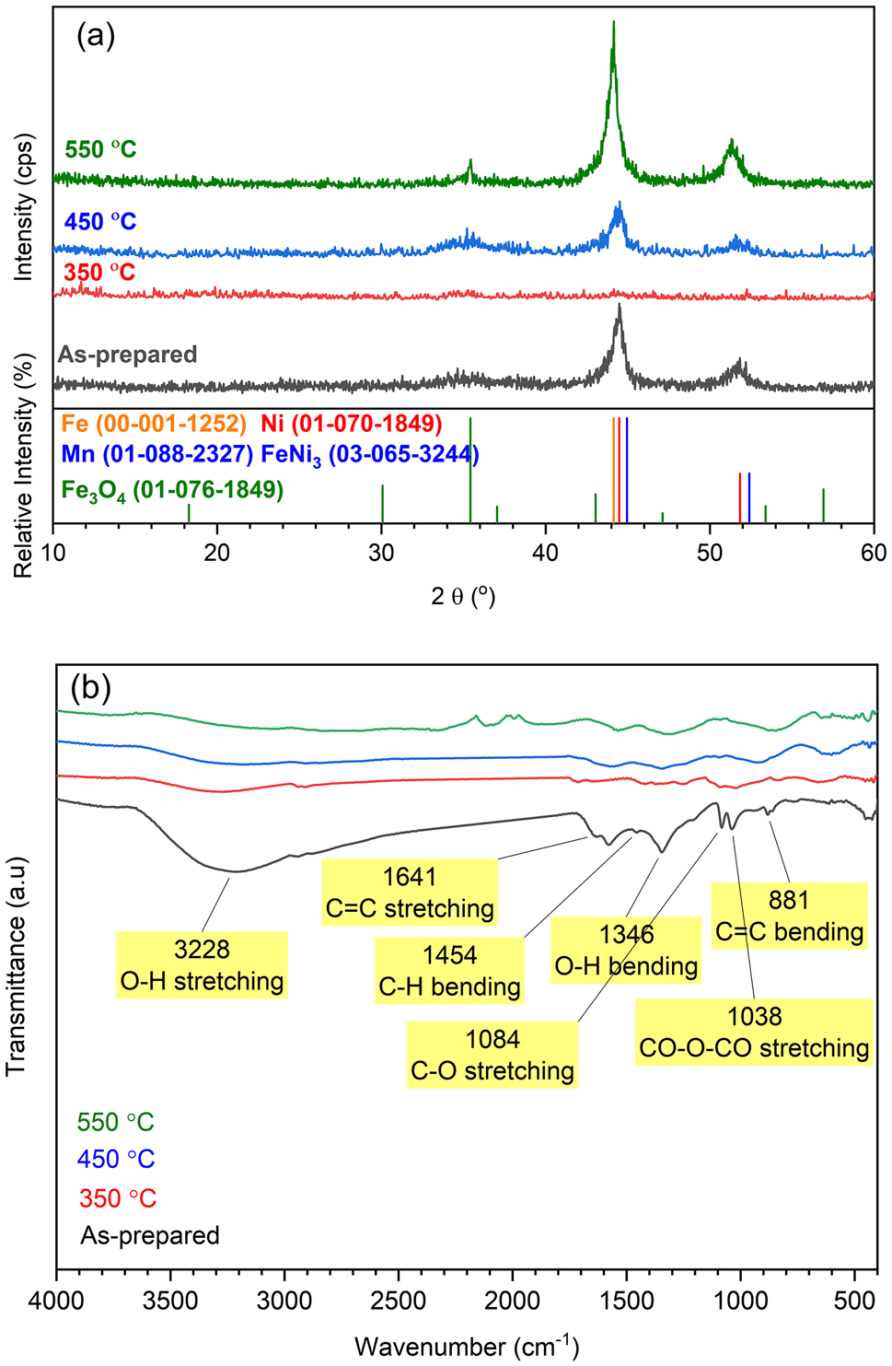
(**a**) XRD patterns, and (**b**) FTIR spectra of the as-prepared alloy as well as the Fe_0.65_Ni_0.30_Mn_0.05_@C (50% PVA) composite prepared at different temperatures.

The properties and adsorption efficiencies of the synthesized materials are significantly influenced by their crystalline phases and functional groups. Therefore, both XRD and FTIR techniques were employed to analyze these characteristics. Figure 2a illustrates the room-temperature XRD patterns of the as-prepared Fe_0.65_Ni_0.30_Mn_0.05_ and the carbon-coated (50% PVA) annealed ternary alloy. The as-prepared sample shows two broad peaks positioned at 2θ = 44.5° and 51.8°. The alignment of the (111) and (200) diffractions of Ni (ICDD 01-070-1849) alongside the (110) reflection of Fe (ICDD 00-001-1252) indicates the presence of a Fe-Ni solid solution, wherein Mn (ICDD 01-088-2327) is integrated into the lattice without exhibiting distinct diffraction characteristics (ICDD 01-088-2327). Those peaks can be attributed to the presence of zero-valent Fe, Ni, and Mn elements, as evidenced by the provided reference patterns, confirming the successful formation of the alloy. Upon heating the alloy with PVA at 350 °C, there was a notable reduction in peak intensities. In prior studies by^28^ and^29^, it was reported that PVA decomposition commences at 300 °C and completes below 500 °C. Consequently, at 350 °C, complete carbonization of PVA may not have occurred, resulting in the presence of partially melted amorphous PVA in the sample and a notably low signal-to-noise ratio in the XRD pattern. Elevating the heating temperature to 450 °C and 550 °C enhanced carbonization of PVA, as indicated by the reappearance of the two diffraction peaks and a heightened signal-to-noise ratio. Significantly, annealing at 550 °C notably improved crystallinity compared to lower annealing temperatures. However, minor peaks associated with the magnetite (Fe_3_O_4_) phase were noticeable, at 2θ = 35.5° and 53.6°, aligning with Fe₃O₄ (ICDD 01-076-1849) XRD data sheet, likely attributable to impurities in the argon gas utilized during calcination. Conversely, the mean size of the grain crystals was determined using Scherrer’s equation^30^. The average grain crystal size was 3.4, 2.2, and 5 nm for samples as prepared, calcinated at 450 °C, and 550 °C, respectively.

The FTIR spectra depicted in Fig. 2b presents the as-prepared samples, revealing distinct bands 3228, 1641, 1454, 1364, 1084, 1038, and 881 cm^− 1^ attributable to the O–H stretching, C = C stretching, C–H bending, O–H bending, C–O stretching, CO-O-CO stretching, and C = C bending^31–33^. These groups are likely derived from residual reagents used in the polyol method during the alloy preparation. Subsequent heat treatment of the alloy in the presence of PVA (at 50%) resulted in the removal of a majority of the functional groups through the carbonization process. Consequently, the heat-treated samples exhibited low hydrophilicity, rendering them suitable for adsorbing weakly polar or non-polar organic compounds such as PAHs.

**Fig. 3:**
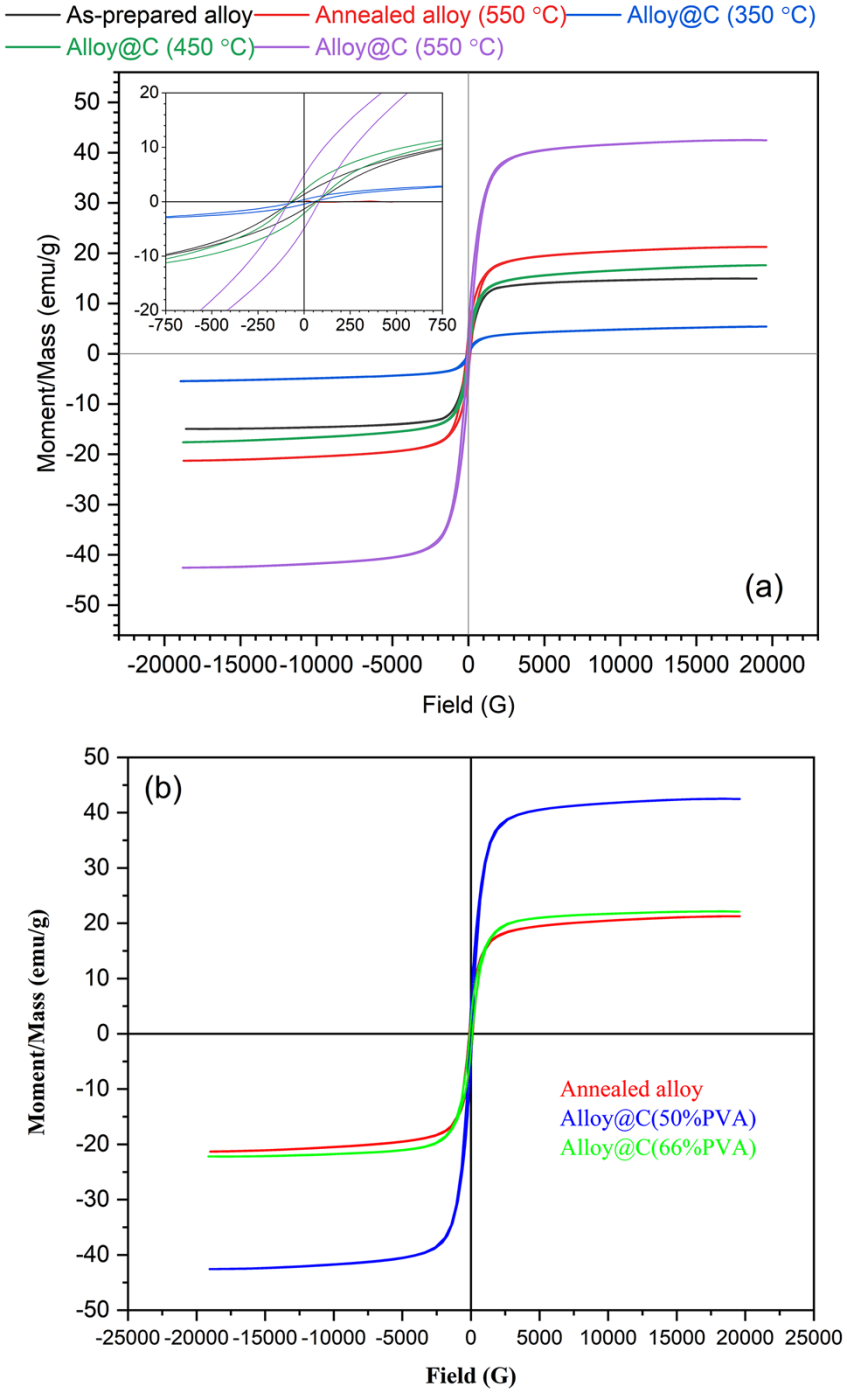
VSM hysteresis loops; effects of (**a**) annealing temperature of 50% of PVA, and (**b**) PVA ratio (annealed at 550 °C).

To ascertain the potential magnetic recovery of the prepared materials, VSM measurements were utilized to appraise the magnetic properties of the synthesized adsorbents, as represented in Fig. 3a. All the samples exhibited a characteristic superparamagnetic behavior due to their low coercivity values of 68, 46, 69, and 82 G for the as-prepared and the composite samples (50% of PVA) annealed at 350 °C, 450 °C, and 550 °C, respectively.

In Fig. 3a, the saturation magnetization recorded an increase with the application of heat treatment. Specifically, it increased from 15 emu/g for the as-prepared alloy to 21 emu/g for the alloy annealed at 550 °C. Among the composite samples, the sample annealed at 350 °C showed the lowest magnetization. Referring back to the XRD data in Fig. 2a, this sample showed a very poor signal-to-noise ratio, revealing the presence of the non-magnetic PVA, which was not completely carbonized at this low annealing temperature, as discussed earlier. The increase in magnetic moment for the annealed samples at 450 °C and 550 °C can be attributed to the development of the alloy crystallinity and the long-range order within the prepared specimen^34, ^as well as the complete carbonization of the PVA. This is consistent with the reported XRD data (see Fig. 2a). Moreover, the improved magnetic properties reflect that the thickness of the carbon layer (@C) and crystallinity in the sample calcined at 550 °C are within an acceptable range that does not reduce the magnetism of the alloy. Surprisingly, the carbon layer enhanced the magnetic properties in the composite sample prepared at 550 °C. This might be due to the complete carbonization of PVA, leaving only a thin layer of carbon around the magnetic particles. This layer prevented the aggregation of magnetic particles and the collapse of the magnetic moment.

In Fig. 3b, the impact of the initial weight% of PVA on the magnetic properties of the composite samples is depicted. The addition of 50% of PVA during preparation increased the magnetic moment to 42.5 emu/g. This is possibly due to the formation of multi-separated domains within the sample as a result of the presence of PVA. These domains interact with each other when an external magnetic field is applied, leading to an enhanced net magnetic moment, as previously reported by Martins and Wurth^35^. However, a higher increase in the PVA amount (i.e., 66%) results in a lower total magnetic moment of 22 emu/g, which is almost the value of the annealed pure alloy. This is because of the high carbon content of the non-magnetic component in this sample. .

**Fig. 4:**
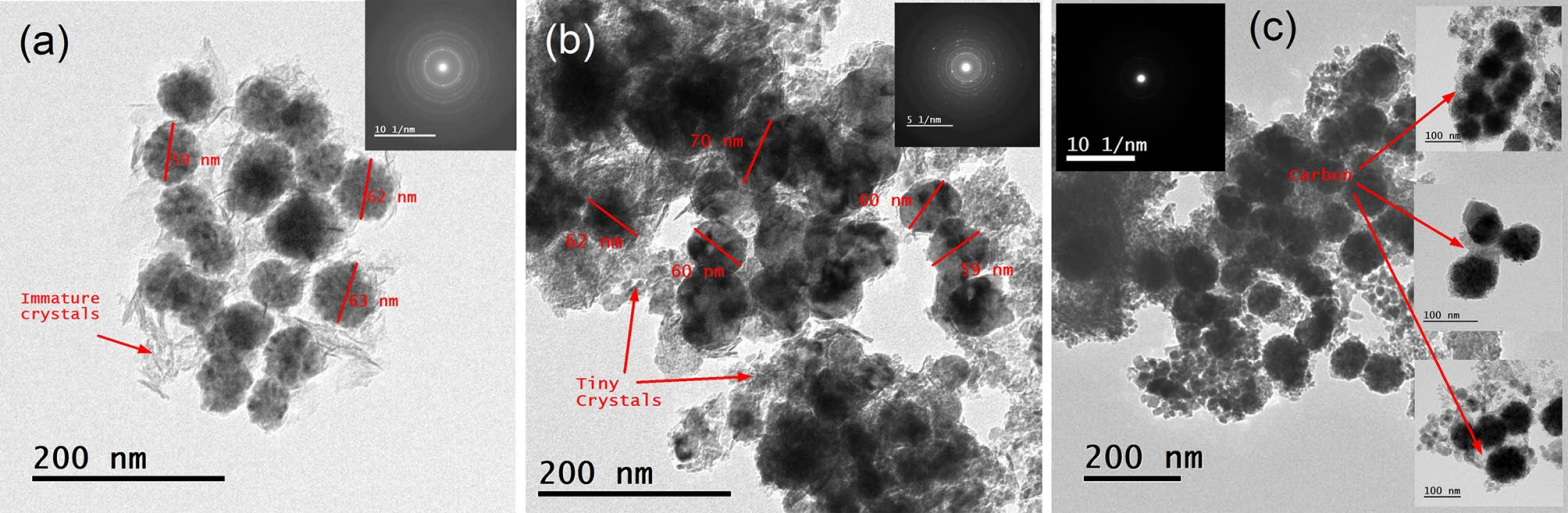
HR-TEM images and SAED patterns for (**a**) the as-prepared alloy, (**b**) annealed alloy at 550 °C, and (**c**) annealed alloy with 50% PVA.

The HRTEM images in Fig. 4a-c show the morphological examinations of the investigated nanostructured pure and modified Invar alloys prepared under different conditions. The images were captured at different magnifications to clearly illustrate the structural features. In Fig. 4a, the as-prepared sample shows a well-assembled group of spherical nanoparticles (~ 60 nm) surrounded by irregular immature crystals. The poorly selected area electron diffraction (SAED) pattern recorded (inset of Fig. 4a) reflects the low crystallinity of the as-prepared sample, which is consistent with the XRD patterns shown in Fig. 2. Annealing the as-prepared sample at 550 °C resulted in fused spherical nanoparticles of almost the same average particle size similar to the as-prepared sample (see Fig. 4b). Additionally, scattered tiny crystals were observed, which might be due to the crystallization of the immature crystals that were observed in the as-prepared samples. Consequently, sharp quasi-rings were observed in the SAED patterns (inset of Fig. 4b), indicating the polycrystalline nature of this sample. Adding PVA in 50 wt% during the annealing step formed a carbon layer around the alloy, as evidenced by the image in Fig. 4c and its inset. Also, the carbonization led to the separation of the alloy spherical particles by the carbon, i.e., fusion of the spherical particles of alloy was inhibited, and this morphology enhanced the magnetization of this sample as noted in the VSM measurement (see Fig. 3b).

**Fig. 5:**
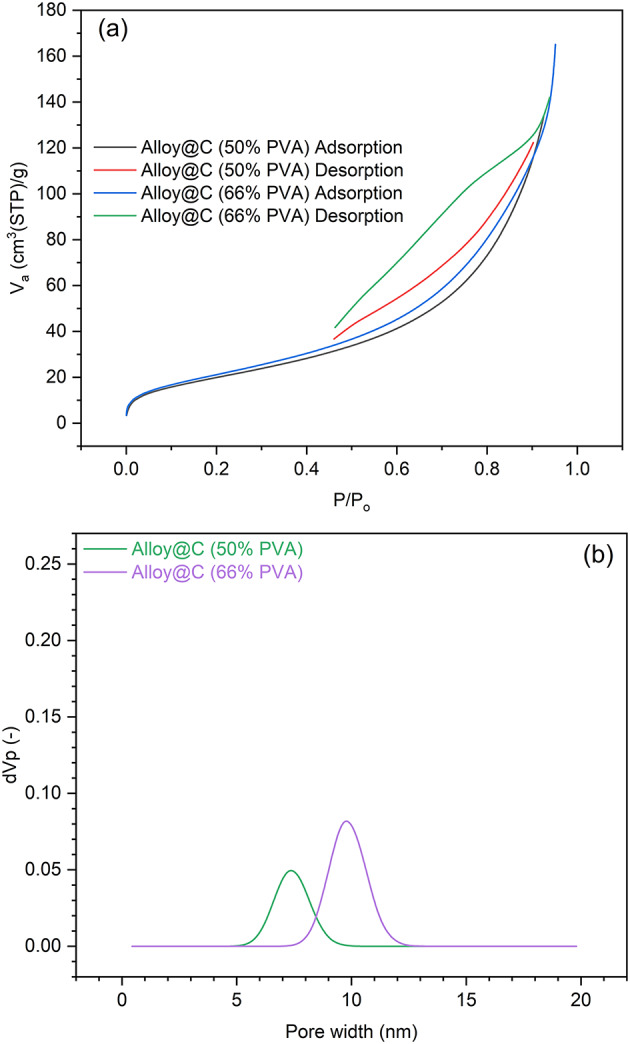
(**a**) N_2_ adsorption-desorption isotherms, and (**b**) pore width distribution of the samples prepared using different initial PVA amounts.

The texture of a material’s surface is an important factor that affects how well the material can adsorb and hold onto other substances. Figure 5a shows the amount of N_2_ gas that the prepared magnetic adsorbent material (alloy@C) can hold at different equilibrium relative pressure (P/P_0_) and porositythe materials pore size distributions. According to IUPAC, six types of physisorption isotherms describe how materials interact with the substances they adsorb. All of the prepared magnetic adsorbents fall into the category of type II isotherm, which is specific to materials that are not porous or have very small pores. This type of isotherm shows that the material can adsorb a single layer of the substance and then start to adsorb multiple layers as the pressure increases. Additionally, all the samples displayed type H3 hysteresis loops, indicating the presence of aggregates (loose assemblages) of platelike particles that form slit-like pores. Based on these observations, it can be deduced that the prepared samples exhibit nearly identical N_2_ adsorption behaviours, regardless of the thickness of the carbon layer. However, the specific surface areas and the calculated pore volume (V_total_) of the alloy@C composites prepared using 50%, and 66% PVA varied from 78 to 84 m^2^/g and 0.1816 to 0.2388 cm^3^/g, indicating that the addition of more PVA results in more available active sites on the adsorbent surface.

When examining the difference in average pore width (Fig. 5b), it was found that using 50% and 66% PVA resulted in the formation of mesopores with large volumes due to the thick carbon layer.

### Adsorption study

**Fig. 6:**
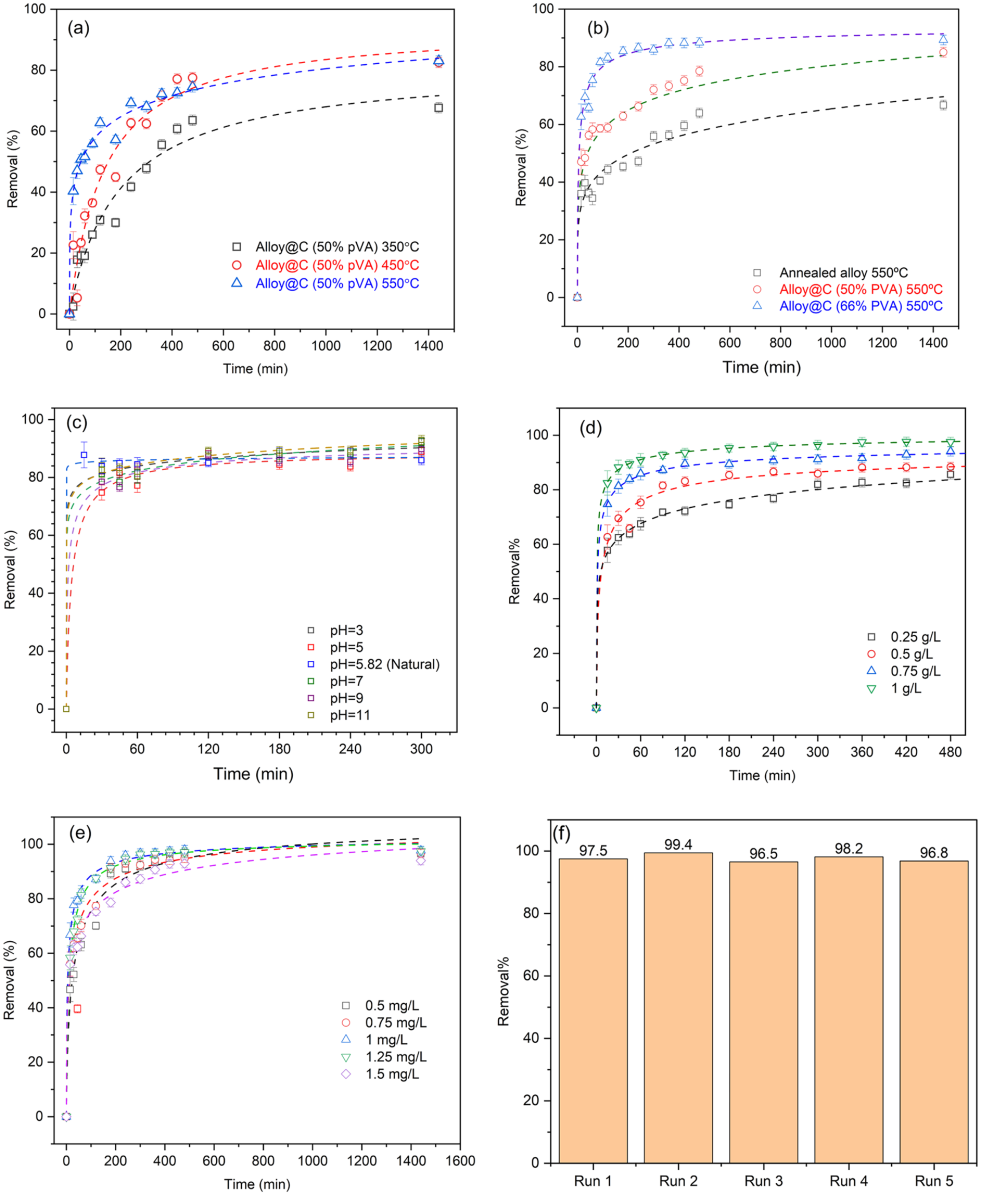
Optimization of PHE removal using the prepared materials; Effects of (**a**) calcination temperature, (**b**) ratio of PVA, (**c**) pH, (**d**) adsorbent dose, (**e**) initial concentration of PHE, and (**f**) reusability test.

Performance analysis of the synthesized magnetic adsorbent materials at varying calcination temperatures, carbon loading quantities on the magnetic core, pH levels, dosages of the magnetic adsorbent material,  initial concentration of phenanthrene, and the reusability test is illustrated in Fig. 6. According to Fig. 6a, Fe_0.65_Ni_0.30_Mn_0.05_@C prepared using 50% PVA annealed at 550 °C, exhibited the highest phenanthrene elimination efficacy.

The total carbonization of PVA render it more hydrophobic than Fe_0.65_Ni_0.30_Mn_0.05_. The incorporation of PVA into Fe_0.65_Ni_0.30_Mn_0.05_ annealed at 550 °C is illustrated in Fig. 6b. The Fe_0.65_Ni_0.30_Mn_0.05_@C using 66% PVA exhibits the highest phenanthrene elimination efficiency. The increase in PVA loading may result in heightened porosity of the manufactured magnetic adsorbents, subsequently enhancing the number of active sites and surface area, as illustrated in Fig. 5b.

Conversely, Fig. 6c illustrates the influence of pH medium on adsorption efficacy, observing the insignificance of phenanthrene’s elimination. This observation can be attributed to the nonpolar nature of both phenanthrene and the carbon-based surface of the synthesized magnetic nanoparticles. Since phenanthrene is hydrophobic and lacks ionizable functional groups, its interaction with the adsorbent is primarily driven by hydrophobic forces rather than electrostatic interactions. Such behavior is consistent with previous studies on the adsorption of non-polar organic pollutants onto carbon-based materials^3^. Figure 6d illustrates the impact of the dosage of synthesized magnetic adsorbents (Fe_0.65_Ni_0.30_Mn_0.05_@C at 66% PVA, annealed at 550 °C) at natural pH. Increasing the quantity of the manufactured magnetic adsorbent enhances and accelerates adsorption efficiency. The elimination of phenanthrene escalated until it attained a concentration of 1 g/L, which effectively removed 99.5% of phenanthrene, demonstrating the exceptional effectiveness of the synthesized material.

To evaluate the impact of phenanthrene initial concentration, only 0.25 g/L of adsorbent was utilised to slowing down the adsorption process and enabling monitoring of changes in the removal. As seen in Fig. 6e, the control over the adsorption process is empowering, with less phenanthrene concentration being adsorbed more quickly, as predicted.

Under the given ideal reaction conditions, the alloy’s reusability was evaluated at natural pH, 1 g/L dose of adsorbent, and 1.5 mg/L phenanthrene. To remove the adsorbed phenanthrene, the adsorbent material in this test was magnetically retrieved. It was then washed with ethanol several times. The adsorbent was then dried and used in the subsequent cycle. Figure 6f presents the findings. After five consecutive runs, it is clear that the performance of alloy@C has mostly stayed the same in adsorption effeciency throughout many uses.

Many of the two-parameter adsorption isothermal models (e.g., Sips, Freundlich, Dubinin–Radushkevich, Temkin, Hill and Langmuir) were examined using nonlinear curve fits as shown in Table 1. These models not only estimate the adsorption efficiency at equilibrium and at constant temperature, but also provide information on the interaction between the adsorbate and adsorbent^36^.

**Table 1:** The explored isothermal and kinetic models with the extracted parameters.

Model	Equation	Parameters	*R* ^2^	χ^2^	Standard deviation (SD)	RSS
Adsorption isotherms						
Sips	$\:{q}_{e}=\frac{{q}_{s}{{(K}_{s}\:{C}_{e})}^{{B}_{s}}}{1+\:{{(K}_{s}\:{C}_{e})}^{{B}_{s}}}$ ^3^,^36^	q_s_ = 1.93 mg/g K_s_ = 12.94 L/g B_s_ = 6.90	0.997	10.7 × 10^− 4^	0.51 1.09 1.70	10.7 × 10^− 4^
Freundlich	$\:{q}_{e}={K}_{F}\:{C}_{e}^{1/n_{F}}$ ^3^,^36^	K_F_ = 11235.61 L/g n_F_ = 0.27	0.987	24.3 × 10^− 4^	8817.02 0.02	48.6 × 10^− 4^
Dubinin–Radushkevich	$\:{q}_{e}={q}_{m,DR}\:{e}^{-{K}_{DR}{\epsilon\:}_{DR}^{2}},\:{\epsilon\:}_{DR}=RT\:\left(1+\frac{1}{{C}_{e}}\right)\:$^3^,^36^	q_m, DR_ = 163.47 mg/g K_DR_ = 1.27 × 10^− 7^ mol^2^/kJ^2^	0.989	19.4 × 10^− 4^	65.40 1.01 × 10^− 8^	38.7 × 10^− 4^
Temkin	$\:{q}_{e}=\left(\:\frac{R\:T}{{b}_{TM}}\right)\:ln\left({K}_{TM}\:{C}_{e}\right)$^3^,^36^	b_TM_ = 66.99 J/mol K_TM_ =17.68 L/g	0.998	3.6 × 10^− 4^	2.07 0.15	7.19 × 10^− 4^
Hill	$\:{q}_{e}=\frac{{qs}_{H}\:{C}_{e}^{{n}_{H}}}{{K}_{D}+{C}_{e}^{{n}_{H}}}$ ^3^,^36^	qs_H_ = 1.93 mg/L K_D_ = 2.12 × 10^− 8^ L/mg n_H_ = 6.90	0.997	10.7 × 10^− 8^	0.54 1.32 × 10^− 7^ 2.46	10.7 × 10^− 4^
Langmuir	$\:{q}_{e=}{q}_{m\:}{K}_{L}\left(\frac{{C}_{e}}{1+{K}_{L}{C}_{e}}\right)$ ^26^	$\:{q}_{m}$= 1954.4 mg/g $\:{K}_{L}$= 0.00456 L/mg	0.37	65.33	1.46 × 10^7^ 34.22	130.67
Kinetics models						
Pseudo-first-order	$\:{q}_{t}={q}_{e}\left(1-{e}^{-{k}_{1}\:t}\right)$^3^,^36^	k_1_ = 0.065 min^− 1^ q_e_ = 2.31 mg/g	0.937	0.02785	0.01 0.05	0.33
Pseudo-second-order	$\:{q}_{t}=\frac{{k}_{2}\:{q}_{e}^{2}\:t}{1+{k}_{2}\:{q}_{e}\:t}$^3^,^36^	k_2_ = 0.049 g/mg/min q_e_ = 2.43 mg/g	0.982	7.97 × 10^− 3^	0.007 0.03	0.0957
Elovich	$\:{q}_{t}=1/{b}_{e}\text{ln}\left(1+{a}_{e}{b}_{e}t\right)$ ^3^,^36^	a_e_ = 201.34 mg/g/min b_e_ = 5.44 g/mg	0.978	9.66 × 10^− 3^	270.01 0.66	0.11589
Weber Morris	$\:{q}_{t}=k{t}^{1/2}+B$ ^3^,^36^	K = 0.04256 mg/g.min B = 1.47316 mg/g	0.41		0.01 0.24	3.11668
Avrami	$\:{q}_{t}={q}_{e}(1-{{e(}^{-{k}_{1}\:t})}^{n}$ ^3^,^36^	q_e_=2.207 mg/g k = 8399 min^− 1^ *n* = 8399	0.85		0.07 0 0	0.77657

To ascertain the operational design of the manufactured material, information on adsorption models is needed. Every model used in this study has a thorough explanation found in the earlier literature^3,37^.

**Fig. 7:**
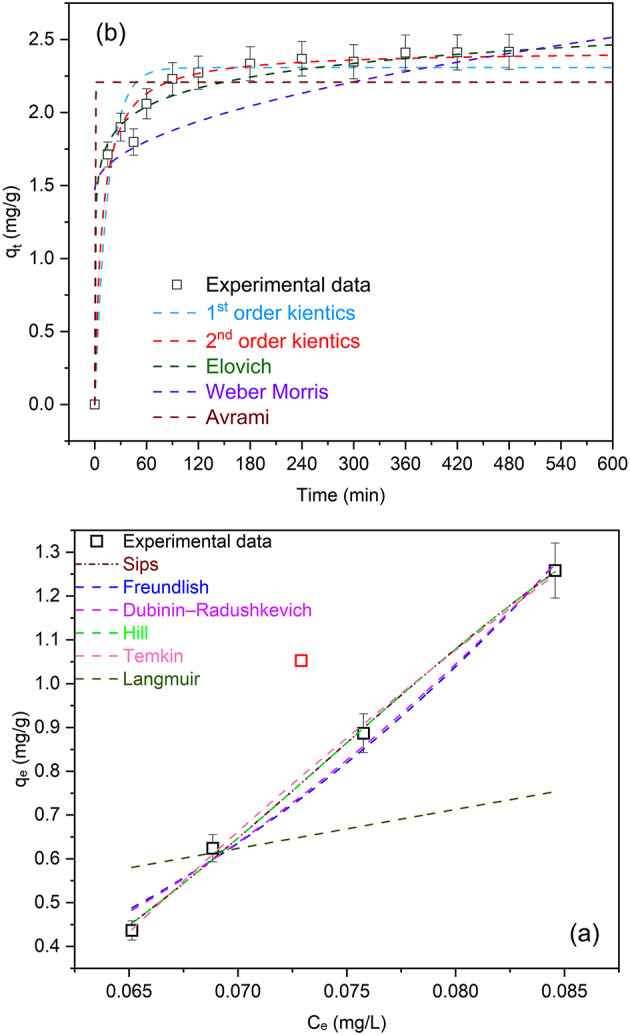
Non-linear (**a**) adsorption isothermal fittings, and (**b**) adsorption kinetics fittings, (C_phenanthrene_ = 1.5 mg/L, pH = 5.8 (natural)).

Several error functions, such as the coefficient of determination (R^2^, the Chi-square (χ2) test, and the residual sum of squares (RSS) statistical error functions, were used to validate each model that was tested. With low values for RSS and χ2, and an R^2^ value that is nearly equal to unity, the Temkin model fits the data the best (Fig. 7a)^36^.

The Temkin model has the capability to examine the connection between indirect adsorbate interactions and the adsorbent. There are multiple layers involved in the sorption process^3^. Because the concentration change was very rapid employing a high adsorbent dosage, as shown in Fig. 6e, the adsorption investigation was conducted at an adsorbent dose of 0.25 g/L to allow follow-up of the variation in adsorbate concentration with time. This perspective diverges from that of the Freundlich model, which focuses on multilayer adsorption occurring on heterogeneous surfaces. The Langmuir model, which posits monolayer adsorption on a uniform surface with a limited number of identical binding sites and no interactions among adsorbed molecules, was utilized in this research. Although the Langmuir model yielded acceptable correlation coefficients, its comparatively lower R² and elevated χ² and RSS values indicate that the adsorption process does not adhere strictly to ideal monolayer behavior. This discrepancy may be due to surface heterogeneity or differences in adsorption energy across the adsorbent surface. The Sips model is also relevant, as it describes adsorption on heterogeneous surfaces without the constraint of infinite adsorption capacity. If the Sips model does not provide a suitable fit, it is worth discussing the reasons, such as the possibility that the nature of heterogeneity is more effectively represented by the linear decline in adsorption heat as indicated by the Temkin model. Additionally, the Dubinin–Radushkevich model is frequently employed to analyze adsorption in micropores and can yield insights into the mean free energy of adsorption, while the Hill model is commonly utilized for scenarios involving cooperative adsorption. The prominence of the Temkin model in our research indicates that the adsorption mechanism is significantly affected by indirect interactions, which may arise from fluctuations in adsorption energy due to surface heterogeneity or alterations in the chemical environment of the adsorbate throughout the adsorption process.

Investigation of the adsorption kinetics is essential for assessing the adsorption process. Figure 7b demonstrates the experimental values of the adsorbed amount ($\:{q}_{t}$) of phenanthrene onto Fe_0.65_Ni_0.30_Mn_0.05_@C at a ratio of 66% PVA, annealed at 550 °C, as a function of contact time. Five kinetic models (pseudo-first-order, pseudo-second-order, Elovich, Intra-particle diffusion model (Weber-Morris), and Avrami) have been used in the nonlinear curve fitting of the collected experimental data. The equations of the five models and their extracted parameters are summarized in Table 1. The adsorption study was carried out at a low adsorbent dose (0. 5 g/L) to allow following up the variation in adsorbate concentration with time because the concentration change was very fast when using high adsorbent dose, as illustrated in Fig. 6e. The linearized Boyd plot did not pass through the origin, suggesting that film diffusion is not the sole controlling step. In contrast, the intraparticle diffusion model exhibited multi-linearity, indicating contributions from both boundary layer and pore diffusion. The condensed parameter displayed in Table 1 demonstrates that second-order kinetics govern the phenanthrene adsorption on Fe_0.65_Ni_0.30_Mn_0.05_@C at a ratio of 66% PVA annealed at 550 °C. This means that chemisorption is the rate-limiting phase and that the adsorption rate depends more on the adsorption capacity than on the adsorbate concentration.

Table S1 presents a comparative analysis of the adsorption capacity (q) under optimal conditions for the Fe_0.65_Ni_0.30_Mn_0.05_@C sample, which was synthesized with a 66% PVA ratio and subjected to annealing at 550 °C, alongside other magnetic carbon adsorbents documented in the literature for the removal of PAHs.

### **Thermodynamics analysis of the phenanthrene adsorption**

The study examined the adsorption of phenanthrene on the Fe_0.65_Ni_0.30_Mn_0.05_@C sample, which contains 66% PVA and was annealed at 550 °C, across various temperatures (25, 30, 35, 40, and 45 °C). This investigation aimed to assess the influence of temperature on the adsorption process and to determine the thermodynamic parameters of adsorption, including free energy (ΔG°), enthalpy (ΔH°), and entropy (ΔS°). The relationship between the equilibrium constant (Kc) and temperature (T) is typically characterized by the Van’t Hoff as follows^27^:

3 $$\:\varDelta\:{G}^{o}=-RT\text{ln}\left({K}_{c}\right)$$

In this context, R (8.314 J/mol K) represents the gas constant. The equilibrium constant, along with the thermodynamic parameters, can be determined through the subsequent equations^27,38–40^.

4 $$\:{K}_{c}=\frac{{C}_{o}-{C}_{e}}{Ce}$$

5 $$\:\text{ln}{K}_{c}=\frac{\varDelta\:{S}_{ads}^{o}}{R}-\left(\frac{\varDelta\:{H}_{ads}^{o}}{R}\times\:\frac{1}{T}\right)$$

6 $$\:\varDelta\:{G}^{0}=\varDelta\:{\text{H}}^{o}-\text{T}\:\varDelta\:{\text{S}}^{o}$$

**Fig. 8:**
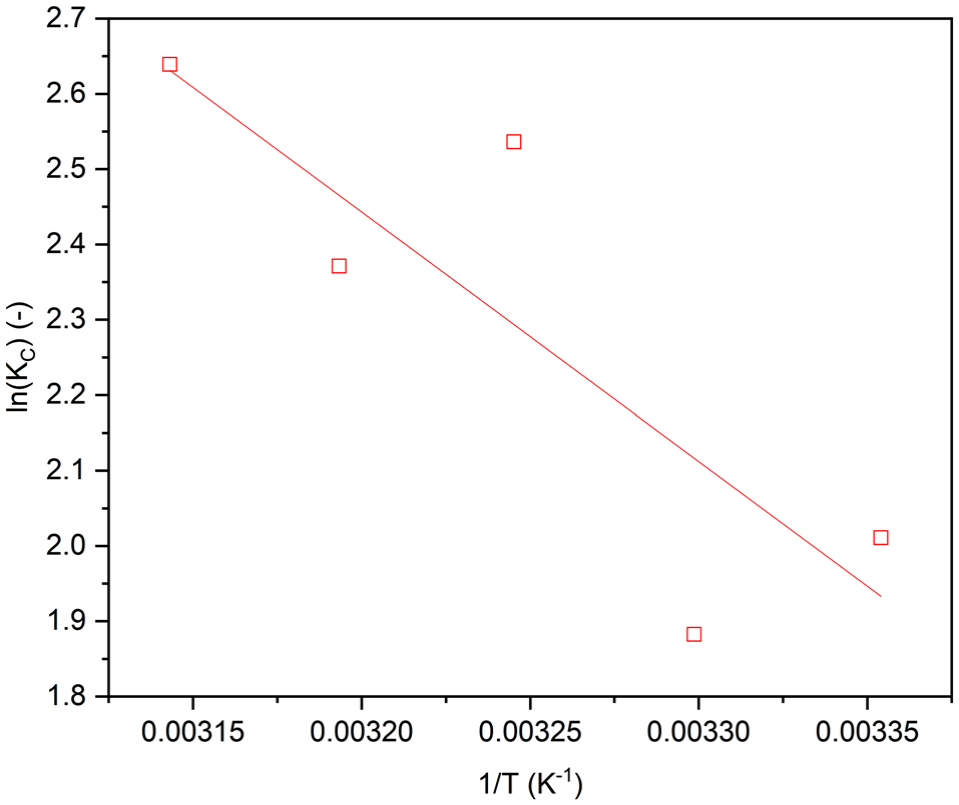
Effect of temperature on the adsorption of phenanthrene on Fe_0.65_Ni_0.30_Mn_0.05_@C, prepared using 66% PVA and was annealed at 550 °C. sample (C_o_ = 1.5 mg/L, dose = 0.5 g/L and pH = natural).

Figure 8 illustrates the relationship between 1/T and temperature, while the calculated thermodynamic parameters are detailed in Table S2. The adsorption of phenanthrene, as analyzed, is characterized as a spontaneous process, indicated by the negative ΔG° values. Furthermore, the observed reduction in ΔG° with rising temperature suggests that the adsorption process becomes increasingly favourable at elevated temperatures. This phenomenon may be attributed to the endothermic nature of the adsorption process, as evidenced by the positive ΔH° values. Additionally, the positive ΔS° values indicate an increase in randomness at the solid–solution interface during the adsorption of phenanthrene onto the active sites of Fe_0.65_Ni_0.30_Mn_0.05_@C, which contains 66% PVA and was annealed at 550 °C.

### Adsorption mechanism

**Fig. 9:**
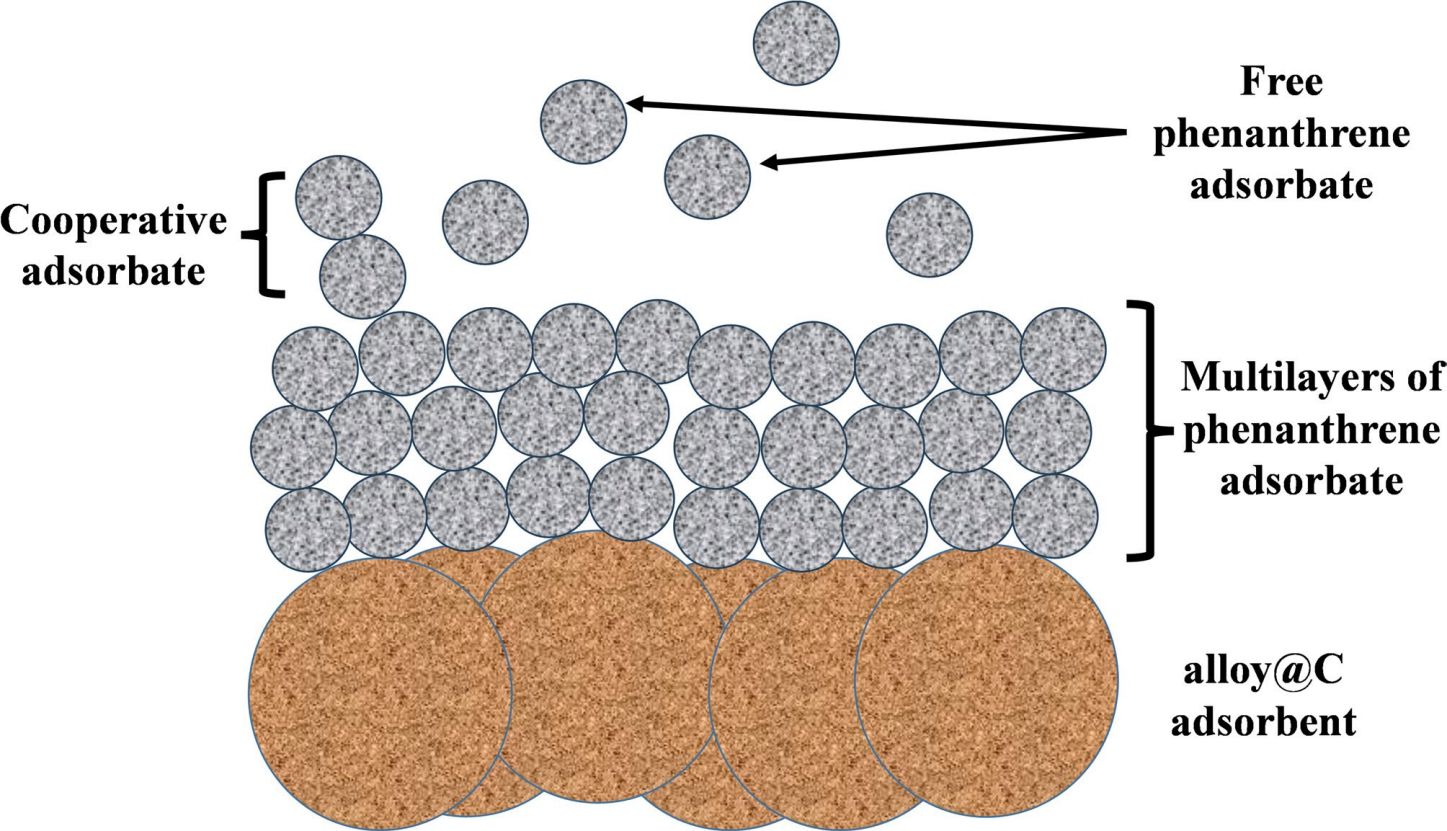
Possible adsorption mechanism between Fe_0.65_Ni_0.30_Mn_0.05_@C and phenanthrene.

The mechanism of phenanthrene adsorption onto Fe_0.65_Ni_0.30_Mn_0.05_@C is predominantly influenced by hydrophobic interactions between the nonpolar aromatic compound and the hydrophobic carbon surface, as illustrated in Fig. 9. This is evidenced by the strong correlation with the Temkin isotherm model, indicating heterogeneous binding on the surface. Additionally, the pseudo-second-order kinetic model suggests that the adsorption process involves several stages, including diffusion and surface interaction. Although the metallic core contributes to the magnetic separability and stability of the material, it does not play a direct role in the adsorption process. These results underscore the composite’s efficacy in eliminating hydrophobic organic pollutants via physical adsorption mechanisms.

## Conclusions

The effective creation of a magnetic core-shell nanostructured Invar alloy Fe_0.65_Ni_0.30_Mn_0.05_@C through a straightforward polyol method illustrates a promising strategy for the development of efficient and recyclable adsorbents for environmental remediation. The superparamagnetic characteristics of the optimized Fe_0.65_Ni_0.30_Mn_0.05_@C (66% PVA) at 550 °C facilitate both easy magnetic separation and stable dispersion in aqueous environments, rendering it highly suitable for water treatment applications. Its remarkable adsorption capability for phenanthrene (99.5% removal) highlights its potential in addressing polycyclic aromatic hydrocarbon (PAH) pollution. The adsorption mechanism, driven by chemisorption as evidenced by the Tekmin isotherm and second-order kinetics, indicates robust interactions between the adsorbent and PAHs. This prepared adsorbent shows strong promise for real-world applications in industrial wastewater treatment, particularly in removing persistent organic pollutants such as PAHs from contaminated water sources. These results contribute to sustainable water purification methods by integrating high efficiency with easy recovery.

## Supplementary Information

Below is the link to the electronic supplementary material.

Supplementary Material 1

## Data Availability

All data generated or analyzed during this study are included in this published article.
