# Supplementary material for: Carbon coated Fe0.65Ni0.30Mn0.05 magnetically separable adsorbent for phenanthrene removal
Source: Sci Rep. 2025 Aug 29;15:31894. doi: 10.1038/s41598-025-13895-3 (PMC12397268; doi:10.1038/s41598-025-13895-3)
Supplement: Supplementary file 1 — Supplementary Material 1 [file 41598_2025_13895_MOESM1_ESM.docx]

Supplementary Information

**Fe_0.65_Ni_0.30_Mn_0.05_@C: A Core@Shell Magnetically Separable Adsorbent for Efficient Water Purification**

Fagr A. Shehata^a^, Mahmoud S. Abdel-Wahed^[[1]](#footnote-1)a^, Mohamed Obaida^b^, Amer S. El-Kalliny^a^, Tarek A. Gad-Allah^a^

^a^Water Pollution Research Department, National Research Centre, 33 El Buhouth St., Dokki, 12622 Giza, Egypt

^b^ Solid State Physics Department, Physics Division, National Research Centre, 33 El Buhouth St., Dokki, 12622 Giza, Egypt


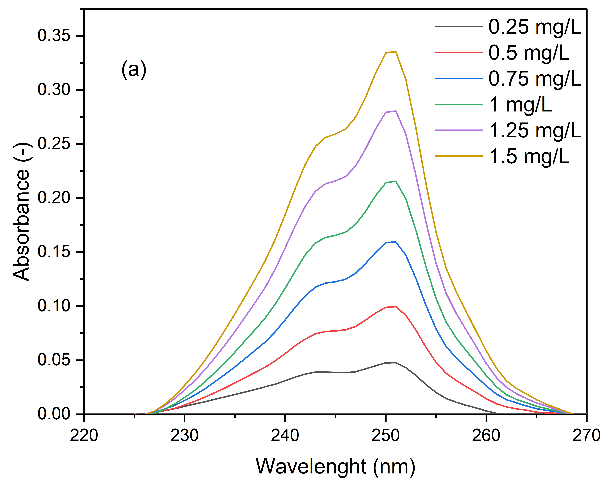

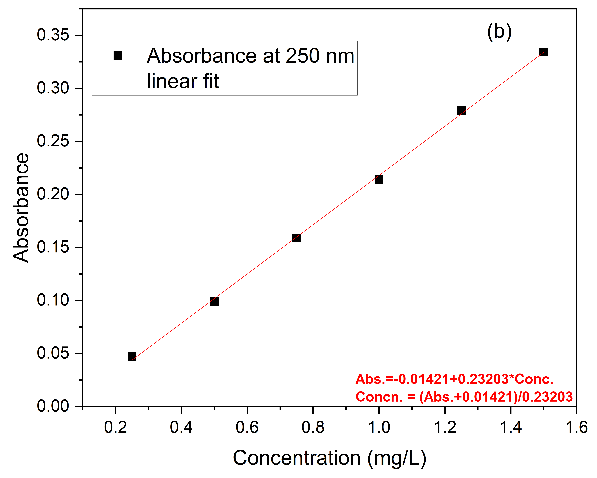


**Fig. S1 Phenanthrene (a) UV-vis spectra, and (b) calibration curve.**

**Table S1. Various magnetic carbon adsorbents for the removal of PAHs**

| **Magnetic adsorbents** | **Adsorbate** | **q (mg/g)** | **M_S_ (emu/g)** | **References** |
| --- | --- | --- | --- | --- |
| Magnetic powder activated carbon (MPAC) | low molecular weight PAHs | 8.74 to 11.37 |  | ^1^ |
|  | high molecular weight PAHs | 8.43 to 20.21 |  |  |
| Magnetically modified rice husk biochar (MBC) | PHE | 97.6 |  | ^2^ |
| Magnetic activated carbon nanocomposite | PAHs | 15.86 | 34.2 | ^3^ |
| Fe_3_O_4_-GO | PHE | 13.65 | 33 | ^4^ |
| Magnetic single-walled carbon nanotubes | PHE | 1.8 | 34.9 | ^5^ |
| Fe_0.65_Ni_0.30_Mn_0.05_@C | PHE | 2.43 | 22 | This work |

**Table S2 Thermodynamic parameters of phenanthrene adsorption.**

| ΔG (J/mol) | | | | | ΔH° (J/mol) | ΔS° (J/mol K) |
| --- | --- | --- | --- | --- | --- | --- |
| 298.15 K | 303.15 K | 308.15 K | 313.15 K | 318.15 K | 27522.26 | 108.38 |
| -0.05607 | -0.05163 | -0.06842 | -0.06295 | -0.06896 |  |  |

1 Mirzaee, E. & Sartaj, M. Activated carbon‐based magnetic composite as an adsorbent for removal of polycyclic aromatic hydrocarbons from aqueous phase: Characterization, adsorption kinetics and isotherm studies. *Journal of Hazardous Materials Advances* **6**, 100083, doi:<https://doi.org/10.1016/j.hazadv.2022.100083> (2022).

2 Guo, W., Wang, S., Wang, Y., Lu, S. & Gao, Y. Sorptive removal of phenanthrene from aqueous solutions using magnetic and non-magnetic rice husk-derived biochars. *Royal Society open science* **5**, 172382 (2018).

3 Inbaraj, B. S., Sridhar, K. & Chen, B.-H. Removal of polycyclic aromatic hydrocarbons from water by magnetic activated carbon nanocomposite from green tea waste. *Journal of Hazardous Materials* **415**, 125701, doi:<https://doi.org/10.1016/j.jhazmat.2021.125701> (2021).

4 Huang, D., Xu, B., Wu, J., Brookes, P. C. & Xu, J. Adsorption and desorption of phenanthrene by magnetic graphene nanomaterials from water: Roles of pH, heavy metal ions and natural organic matter. *Chemical Engineering Journal* **368**, 390-399, doi:<https://doi.org/10.1016/j.cej.2019.02.152> (2019).

5 Zhang, J., Li, R., Ding, G., Wang, Y. & Wang, C. Sorptive removal of phenanthrene from water by magnetic carbon nanomaterials. *Journal of Molecular Liquids* **293**, 111540 (2019).

1. Corresponding author: Mahmoud S. Abdel-Wahed; email: [ms.abdel-wahed@nrc.sci.eg](mailto:ms.abdel-wahed@nrc.sci.eg); Tel.: +201007662985. [↑](#footnote-ref-1)
